# Addressing social needs in oncology practices: A case study of a patient-centered approach using health information technology

**DOI:** 10.1017/cts.2024.570

**Published:** 2024-09-30

**Authors:** Helen M. Parsons, David Haynes, Anne Blaes, Timothy R. Church, Julia Halberg, Steven G. Johnson, Pinar Karaca-Mandic

**Affiliations:** 1 Division of Health Policy and Management, School of Public Health, University of Minnesota, Minneapolis, MN, USA; 2 Masonic Cancer Center, Minneapolis, MN, USA; 3 Institute for Health Informatics, University of Minnesota, Minneapolis, MN, USA; 4 XanthosHealth, Minneapolis, MN, USA; 5 Division of Hematology, Oncology and Transplantation, University of Minnesota, Minneapolis, MN, USA; 6 Division of Environmental Health Sciences, School of Public Health, University of Minnesota, Minneapolis, MN, USA; 7 Carlson School of Management, University of Minnesota, Minneapolis, MN, USA

**Keywords:** Social determinants of health, community based organizations, oncology practices, patient-centered design, mobile health information technology

## Abstract

Given the dramatic growth in the financial burden of cancer care over the past decades, individuals with cancer are increasingly susceptible to developing social needs (e.g., housing instability and food insecurity) and experiencing an adverse impact of these needs on care management and health outcomes. However, resources required to connect individuals with needed social and community services typically exceed the available staffing within clinical teams. Using input from focus groups, key informant interviews, user experience/user interface testing, and a multidisciplinary community advisory board, we developed a new technology solution, ConnectedNest, which connects individuals in need to community based organizations (CBOs) that provide services through direct and/or oncology team referrals, with interfaces to support all three groups (patients, CBOs, and oncology care teams). After prototype development, we conducted usability testing, with participants noting the importance of the technology for filling a current gap in screening and connecting individuals with cancer with needed social and community services. We employ a patient-empowered approach that engages the support of an individual’s healthcare team and community organizations. Future work will examine the integration and implementation of ConnectedNest for oncology patients, oncology care teams, and cancer-focused CBOs to build capacity for effectively addressing distress in this population.

## Introduction

Although cancer mortality rates have declined in the US in recent decades, patients experience differential access to advances in cancer prevention, early detection, treatment, and survivorship care [1]. While prior research has demonstrated that multiple factors (e.g., genetic, environmental, and behavioral) contribute to cancer risk and survival after diagnosis, social needs (e.g., lack of social connection, transportation difficulties, and housing instability) largely shape the health and well-being of individuals, create health inequities, and drive a large fraction of avoidable adverse health outcomes and healthcare costs for these individuals [2,3].

Cancer patients are at particular risk for increasing social needs given the dramatic growth in the financial burden of cancer over the past decades [4,5]. The annual cost of many new cancer drugs now exceeds $100,000 [6], and health insurers are increasingly shifting the cost of cancer care to patients through higher out-of-pocket costs. At the same time, providers are increasingly time- and resource-constrained to assist patients with social needs that extend beyond medical care [7]. As a result, individuals with cancer continue to experience poor access to services that address these social needs, including issues with financial hardship, food and housing insecurity, and transportation difficulties, among other challenges [8]. Previous research has demonstrated significant financial hardship among cancer survivors (e.g., debt, bankruptcy, and delaying care because of cost), with some studies reporting more than 50% of the population experiences financial strain [9]. As a result of this increased financial strain, cancer patients are more likely to experience food insecurity compared to the general population, including problems with food running out and eating unbalanced meals [10,11]. Financial challenges also result in reduced access to reliable transportation, a critical barrier to healthcare access for individuals with cancer, which can contribute to missed medical appointments and poorer health outcomes [12].

Traditionally, connecting individuals with resources to address social needs has fallen to patient navigators and social workers within the oncology care teams. However, resources needed to connect individuals with social and community services typically exceed available staffing of clinical teams [7]. Identifying solutions to build capacity for more effectively connecting oncology patients with needed social and community resources to alleviate distress will benefit patients and providers.

Significant gaps in evidence also remain around best practices for ameliorating social needs. One method to address these needs at an individual level is to screen for social needs, and, among those with identified needs, connect individuals to supportive services through use of health information technology. In many cases, information about social needs is highly private for individuals, especially for those in vulnerable situations. An increasing concern is that collecting social needs poorly can result in trauma, stress, and even legal implications for patients at risk [13–15]. As a result, effective solutions to support the National Comprehensive Cancer Network and Commission on Cancer guidelines for distress screening and the proposed policies by the Centers for Medicare and Medicaid Services to incorporate screening and identification of social needs into quality reporting programs are critically needed [16–19]. The landscape of health information technology to screen and identify resources for addressing social needs is growing, including platforms such as FindHelp [20] and Unite Us [21]. However, these platforms do not create curated, real-time resource connections for patients and often do not include a patient interface; rather, they provide a list of services. Further, these organizations are not tailored to the unique needs and experiences of cancer survivors and do not comprehensively integrate the screening and connection process across key stakeholders (e.g., oncology patients and caregivers, oncology care teams, and community based organizations [CBOs]) in a patient-centered approach.

To address this gap, our team developed an innovative electronic health record (EHR)-enabled health information technology solution to screen, assess social needs, and connect individuals who have cancer to CBOs that can address their needs with the overall goal of improving the health and psychosocial outcomes of these individuals. In this report, we describe our iterative approach leveraging user-centered participatory design techniques to first gather information from a robust set of multidisciplinary stakeholders (oncology patients and caregivers, oncology care teams, and CBOs) and use these insights to develop a new patient-centered technology, ConnectedNest.

## Materials and methods

### Prototype development

#### Stakeholder design input

We utilized a multi-stakeholder design process to develop a new technology for screening individuals with cancer and connecting them to needed services and support. This study was approved by the University of Minnesota Institutional Review Board. Results were reported according to the Consolidated Criteria for Reporting Qualitative Research guidelines (COREQ) [22].

#### Focus groups and key informant interviews

In December 2021, we conducted five virtual focus groups of individuals with cancer and their caregivers across a diverse set of cancers and socioeconomic backgrounds. Participants were recruited via convenience sampling from a multispecialty academic health center in the Midwest. Participants included adults on active treatment or in survivorship as well as their caregivers (with or without the survivor) and were approached in-person at the time of a scheduled oncology visit between November and December 2021 by study staff after a warm handoff from their oncologist. All individuals who initially met eligibility criteria, did not opt out of research, and were interested based on the warm hand off from their oncologist were approached for recruitment. Participants were provided with an informational flyer containing the study purpose, eligibility criteria, and staff contact information. After confirming eligibility, focus groups were scheduled by study staff. We received verbal consent prior to initiation of the focus group for all participants. All five single-session focus groups, which lasted approximately 45-60 minutes each, were conducted in English, online via Zoom, after which participants received a $25 gift card. Focus groups were not stratified by participant demographics. The focus groups were conducted by two female PhD researchers who self-identified as non-Hispanic White and had no prior established relationship with the participants. During the focus groups, participants were asked to reflect on the following topics: 1) nonmedical services and supports that would have been useful after cancer diagnosis, 2) challenges and preferences for accessing nonmedical services, and 3) how to design a system to connect to those resources. Discussion guides were developed based on factors shown to impact social determinants of health as outlined in the World Health Organization Commission on Social Determinants of Health conceptual framework for action [23].

From December 2021 to April 2022, we additionally conducted key informant (KI) virtual interviews with members of the oncology care team from across multiple health systems across the Twin Cities. Participants were identified via snowball sampling based on professional oncology contacts of the study team. Among participants, at the conclusion of the interview, we asked if they could identify other eligible colleagues who met our study inclusion criteria who we then reached out to for additional interviews. KI interviews were conducted individually with the participants and researchers who also conducted the focus groups. One-time interviews lasted 45–60 minutes and participants received a $100 gift card. During the interviews, KIs were asked to reflect on two key topics 1) current processes and tools for screening and connecting individuals with cancer to needed services and supports, 2) and key elements of a novel tool or process that would ensure patients had access to needed services and supports.

For both focus groups and KI interviews, we recorded the content using digital audio recording via Zoom, which was then transcribed by the research team after each interview, de-identifying all datasets and extracting demographic characteristics of each study participant. Study team members Parsons and Hillmer then independently coded transcripts to develop themes for each of the key topics discussed above (see Supplemental Tables 1–2 for a copy of the focus group and interview guides). We conducted our analysis based on the approach outlined in Braun & Clarke’s thematic analysis [24]. We determined our study sample size for focus groups and KI interviews based on theoretical saturation [25], after which the study team concluded that no new data or themes were identified from additional qualitative data collection. Sample size was not determined a priori as it is contingent on evolving themes [26]. We then used a constant comparative approach [27,28] in the context of cancer treatment and survivorship to identify initial key themes within topics. After reviewing the transcripts, we used an iterative process of reducing the data by first using data from our preliminary coding to develop initial themes from the first round of data reduction and then further reducing and recoding until core themes were developed within a topic [28]. Exemplary quotes representing each theme were then selected.

### Advisory board consultation

After themes were identified, we brought the findings to our multidisciplinary Community Advisory Board (Supplemental Table 3) consisting of 12 community based organizations focused on cancer patients to review themes from the focus groups and KI interviews and collect additional context and feedback on developing a prototype technology that screens and connects individuals with needed services and supports after a cancer diagnosis. We then used the combination of themes developed from the KI interviews and focus groups and emphasized by the advisory board to identify key components that were developed and included in the functionality of the prototype application.

### Creating the prototype

We employed a Human Centered Design (HCD) [29] approach while developing our prototype, as it allows software teams to create a personalized interface when designing interfaces for stakeholders. HCD involves users throughout the entire design process, which improves the usability and user experience. We designed and developed a patient-centered social-care referral platform that allowed for unique interfaces designed for the workflows of our stakeholders (i.e., patients, oncology care team, and CBOs). The final platform integrated each of the key themes identified through our focus groups and KI interviews into the core functionality (see key themes outlined in results below). The initial prototype was completed in April 2021 with ongoing input from the study team, a collaboration between the University of Minnesota and XanthosHealth. Details for the prototype, ConnectedNest (a product of XanthosHealth), are also outlined below in the results. Prior to completing the prototype, we consulted again with the Advisory Board to gain additional feedback for refinement of the prototype prior to prototype testing.

### Prototype testing

#### Usability testing

**Figure 1.**
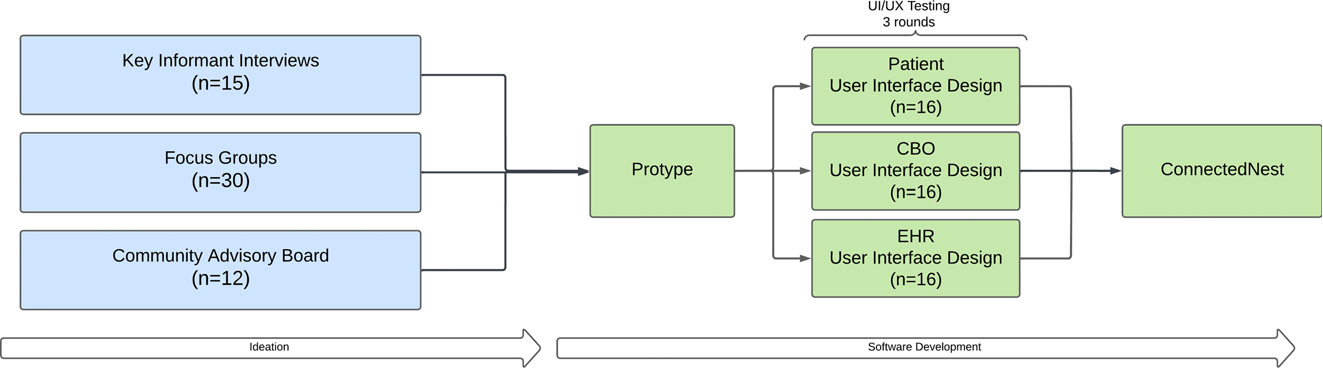
Overview of the process for the development and testing of ConnectedNest. CBO = community based organization; EHR = electronic health record.

After prototype development, the team performed a virtual task-based usability assessment in April 2021 with 48 total cancer patients and caregivers, oncology care teams, and CBO administrators (16 from each group). Each product evaluation session began with a participant briefing, which included an introduction of a think-aloud protocol and an explanation of the procedure for sharing their screen over Zoom. After the briefing was finished, the participant was asked to complete a series of basic tasks (e.g., registration, screening, and connection) based on their user type (patient/caregiver, oncology care team, and CBO administrator) using hypothetical data based on their experience. After each evaluation session, the team recorded issues identified by the participant and agreed on an estimated degree of impact, defined as the user’s inability to complete a task successfully or issues related to user frustration, confusion, or inefficiency. After all sessions had been completed, using a consensus approach, the team discussed each identified issue to modify prior to implementation in health system and patient settings. See Fig. 1 for an overview of the prototype development and testing process.

## Results

### Prototype development

#### Focus groups

Thirty participants took part in five focus groups. Participants included individuals aged 18+ who were patients or caregivers of those receiving cancer or survivorship care within our community and academic clinics in a large integrated health system in Minnesota. Among participants, 13.3% were caregivers and 86.7% were individuals with a history of cancer. The median age was 57 and participants were diverse by cancer type (e.g., breast, prostate, and melanoma), sex, and race/ethnicity (10% Black/African American; 3.3% American Indian; 86.7% White; and 6.7% Hispanic/Latino). Most participants were within five years of diagnosis and their stage of cancer at time of diagnosis ranged from 0 to IV (Table 1). Forty-seven percent of participants had>4 members in their household, with half of participants reporting a median household income <$100,000.

**Table 1.** Characteristics of focus group and key informant interview participants informing development of ConnectedNest

Focus group participants (N = 30)	
Characteristics	N (%)
Role	
Patient	26 (86.7%)
Caregiver	4 (13.3%)
Cancer Type	
Breast	18 (60.0%)
Prostate	2 (6.7%)
Colorectal	1 (3.3%)
Melanoma	1 (3.3%)
Kidney	3 (10.0%)
Non-Hodgkin’s Lymphoma	1 (3.3%)
Other Cancer Type	4 (13.3%)
Cancer Stage at Diagnosis	
0	1 (3.3%)
I	4 (13.3%)
II	5 (16.7%)
III	7 (23.3%)
IV	8 (26.7%)
Other/Unknown	5 (16.6%)
Year of diagnosis	
Prior to 2015	9 (30.0%)
2016-2019	7 (23.3%)
2020-2021	14 (46.7%)
Median Age	57
Gender	
Male	7 (23.3%)
Female	23 (76.7%)
Race	
White	26 (86.7%)
Black	3 (10.0%)
American Indian	1 (3.3%)
Hispanic/Latino	
Yes	2 (6.7%)
No	28 (98.3%)
Median Income	$100,000
Numbers Family Members in Household	
1	3 (10%)
2	12 (40%)
3	1 (3%)
>4	14 (47%)

Key Informant Interview Participants (N = 15)	
Role	
Community Based Organization (CBO)	11 (73.4%)
Oncologist	2 (13.3%)
Social Worker	2 (13.3%)
Gender	
Male	3 (20%)
Female	12 (80.0%
Primary Type of Support Addressed by CBO (N = 11)	
Food Insecurity	1 (9.0%)
Legal and Financial Issues	2 (18.2%)
Social Support and Education	5 (45.6%)
Advocacy and Connection	1 (9.0%)
Multiple Types of Support	2(18.2%)

We developed several key themes from focus group interviews (Table 2). The first theme we identified was that participants have a broad range of needs that include financial help (e.g., money for rent), transportation, housecleaning, help preparing food for patients and their families, help picking up supplies, childcare, and emotional and mental health support. Our second theme highlighted that significant barriers exist to connecting patients with needed resources, with the most notable barrier being lack of easy access to and availability of information on needed social and community services. Finally, we identified a theme regarding how best to design a system to connect individuals to nonmedical services after diagnosis. In that theme, participants noted the need to develop a tool that was not just “a binder of information,” but a resource that would evolve with the changing needs of cancer survivors, provide easy access to contacts from a curated set of engaged organizations, and be linked to a trusted person or organization to introduce or advocate for the technology.

**Table 2.** Key themes identified from focus group participants describing nonmedical services and supports most useful after a cancer diagnosis and systems to connect to these services

Theme	Description of themes and exemplary quotes
Nonmedical services and supports that would have been useful after a cancer diagnosis	
*Theme 1. A broad range of nonmedical services and supports are useful after diagnosis*	Examples Include: Financial Support (e.g., Money for rent) Transportation Housecleaning services Food preparation (e.g., including special dietary needs) Help with errands such as picking up supplies Childcare Emotional support Mental health support Legal services Organizational assistance (e.g., making appointments, renewing prescriptions) Exemplary Quotes Quote 1: “I could really use transportation and my insurance doesn’t cover it” Quote 2: “Help with housecleaning because I have no energy to clean my house” Quote 3: “We don’t have anyone who can come over and babysit when I’m not supposed to be lifting [my kids]. So I had to pick [them] up today. I have to do things I’m not really supposed to be doing, because we don’t have that extended care” Quote 4: “It’s so scary times when you have to make a decision like okay here’s one more bill, but I’ve had to take time off work to go take care of the issue…it can be lonely”
Challenges and preferences for accessing nonmedical services after a cancer diagnosis	
*Theme 2. Significant barriers exist to connecting with needed social and community services after diagnosis.*	*Sub-theme 1: There is a lack of easily accessible and available information on services offered.* Exemplary Quotes Quote 1. “I really struggled to find resources- I know I have a social worker…but I haven’t actually been able to get her to reach out to me so that’s been a huge pain point’ Quote 2: “Nothing was really offered to me, …so it took me a lot of my own research to figure out how to get help… I felt like they pretty much said here’s your cancer here’s your diagnosis and then I was on literally bedridden… I felt very alone.” Quote 3: “You don’t know what to ask for… It’d be nice to know what services are offered because I wasn’t given any of that…It’s just if you need something, let us know.” *Sub-theme 2. There is a missing link between needs and availability of services* Exemplary Quote Quote 4: “[The few recommended services] were booked for months out or..unavailable…or too far for me to drive or didn’t work with my schedule’
How to design a system to connect to nonmedical services after a cancer diagnosis.	
*Theme 3. Participants would like a resource that is adaptable to their treatment and survivorship needs over time and easily links to available needs through a trusted resource*	*Sub-theme 1: Participants do not just want a “binder of information”* Exemplary Quote Quote 1: “When I first got diagnosed I got..this big giant vinyl notebook. And I never looked at it quite honestly…because when you first get that notebook that’s not really when you need all the services..[when] your service requirements change..I was personally so deep into treatment…that it was exhausting to start looking around for it” *Sub-theme 2. Participants want a resource that evolves with changing needs.* Exemplary Quote Quote 2: “It would be nice to have something like an app…that you could check in and say how you are doing today..[and know about resources] typically at your stage or where you are in your cancer treatment” *Sub-theme 3. Having the contacts easily accessible is a priority.* Exemplary Quote Quote 3: “[I was] on the phone all the time trying to get in touch with people…so if there would be some way of…the people getting in touch with us..some way your name would go to all of these services and they could contact you that would be helpful” *Sub-theme 4. The resources needs to be linked to or referred by a trusted person or organization* *Exemplary Quotes* Quote 4: “As long as it comes from a trusted source and I know what’s going to come, I have no problem” Quote 5: “You want to feel comfortable letting someone know that you need some support”

#### KI interviews

Fifteen KIs from oncology care teams (e.g., oncologists and social workers) across multiple health systems and leaders of a diverse set of CBOs (e.g., legal care, social support, and food insecurity) took part in structured interviews (Table 1). Several key themes were developed as part of these interviews (Table 3). First, when discussing the current process and tools for screening and connecting individuals with cancer to needed services and support, KIs noted a shortage of social workers who can provide information on these services. They also identified a lack of consistent screening for nonmedical needs within the medical system. Once identified, health systems and CBOs noted difficulty in meeting the needs of non-English-speaking clients and those from diverse cultures. Finally, they noted use of different tools and screening processes across organizations resulting in duplication of information collected. In order to support more streamlined screening and connection for needed social and community services, KIs identified a number of key areas for development. Themes included that, ideally, there would be a tool that can be introduced before and/or after a clinic visit and by a trusted source. It should be patient-directed as some needs are sensitive to discuss. It would also be beneficial if the tool could interface with the EHR and provide personalized referrals. Finally, there should be a way to remind patients and families, over time, that support is available.

**Table 3.** Key themes from key informant interviews with oncology care team members and community based organizations for developing tools or processes for connecting cancer patients with services to address social needs

Theme	Description of themes and key quotations
Current process and tools for screening and connecting individuals with cancer to needed services and supports	
*Theme 1. The health system has a shortage of providers who can provide patients with information on needed services and supports*	*Sub-theme 1. The primary way patients are connected to services and supports through the health system after diagnosis is through the social work team, but they are under-resourced while patient needs are growing at the same time.* Exemplary Quotes Quote 1: “Social work resources are limited…and staff is already pretty overwhelmed.” [social worker] Quote 2: “The number of social workers that we have in the system is not currently meeting the level of distress that there is system wide” [social worker] Quote 3: “It is really frustrating to be so dependent on the clinics for people to find us.” [community based organization] Quote 4: “Take it off the plate of the clinician…We just have so many things we try to talk about an hour and a half hour visit…going through every service…I don’t I just don’t have time so finding ways to get that out to patients is important.” [oncologist]
*Theme 2. There is a lack of consistent screening and connection for nonmedical needs within the medical system, which can create frustration across stakeholders and duplication of information collected.*	*Sub-theme 1. Lack of consistent process for screening and connection for nonmedical needs in the medical system* Quote 1. “There are a lot of ways that [patients] get connected…I get a lot of referrals to my social work team like nurse coordinators…Oftentimes our schedulers will also reach out to me” [social worker] Quote 2: We don’t have a direct handoff to an agency, it’s more like here’s your resources and then it kind of is up to them to follow through with those resources. And unless there’s like a specific reason…Then it really becomes sort of their follow through.” [social worker] Quote 3: “We rely on them to refer their patients to us and the patient actually has to ask for the service so it’s complicated because we rely on the nurse navigators and the social workers that each of our partner hospitals to remember, along with everything else.” [community based organization] *Sub-theme 2. There is a lack of an actionable questionnaire to determine nonmedical needs, including cultural and language needs, that does not duplicate information already collected.* Quote 3: “We have our own because the [screening tools]…fall woefully short…and we have worked with a lot of the clinics in encouraging them when they do their screening to build out a more robust and meaningful questionnaire” [community based organization] Quote 4: “Sometimes that clinic doesn’t necessarily have the appropriate staff to manage the [patient] because of [their] cultural differences… Language, structural barriers, fear. You name it. It’s really challenging.” [oncologist]
Key elements of a novel tool or process that would ensure patients had access to needed services and supports.	
*Theme 1. Oncology care teams and community based organizations want a patient-centered, adaptable tool that can interface with the electronic medical record and provide personalized referrals.*	*Sub-theme 1. Introduction to available resources should be patient-centered and not rely solely on meeting with a social worker to identify resources.* Quote 1: “There’s some pretty major categories that people with cancer struggle with, and I think it would be important for it to be really easy for them to see [resources ] that they can click and find. In those categories, the things that might help them in the moment… [or] even if they don’t need it right now, that there is help for that.” [community based organization] *Sub-theme 2. Ability to provide personalized referrals, either patient or provider directed* Quote 2: “It would be so [important] if there was a system where I could go in and here’s all the resources and I could just click and it would send them automatically to the patient… Then patients could also pick their own things, of course, but then we could see [who] was actually connected.” [social worker] *Sub-theme 3. Consider having the tool introduced by a trusted member of the care team.* Quote 3: Consider ways that [tools] could help connect or enable people to feel more empowered to realize that there is a provider in a care team that thinks a lot about…the additional support to help people get through treatment..[but] have it available kind of connected with their medical care.” [social worker]

### Community advisory board

Using information from the focus groups and interviews, we brought key themes to our Community Advisory Board. These themes aligned with the experiences of our Advisory Board and were endorsed as key areas of focus when developing our prototype. Once the initial prototype was developed, we again consulted with our Advisory Board prior to prototype testing. The initial reactions to the prototype were overwhelmingly positive, with several noting the need for services and technology such as this to improve the current system challenges. The CAB noted additional considerations for the team to remain mindful of throughout the piloting process including how information would flow between entities, differences in confidentiality needs for specific information (e.g., legal), and the goal of minimizing the reporting burden (e.g., duplicate information on eligibility reported to multiple organizations) for patients and caregivers.

From each of these three sets of stakeholders, we identified key challenges with the current system for connecting with needed social and community services and focused on these needs in developing our final prototype for a novel social-care referral technology, ConnectedNest [30].

### ConnectedNest

**Figure 2.**
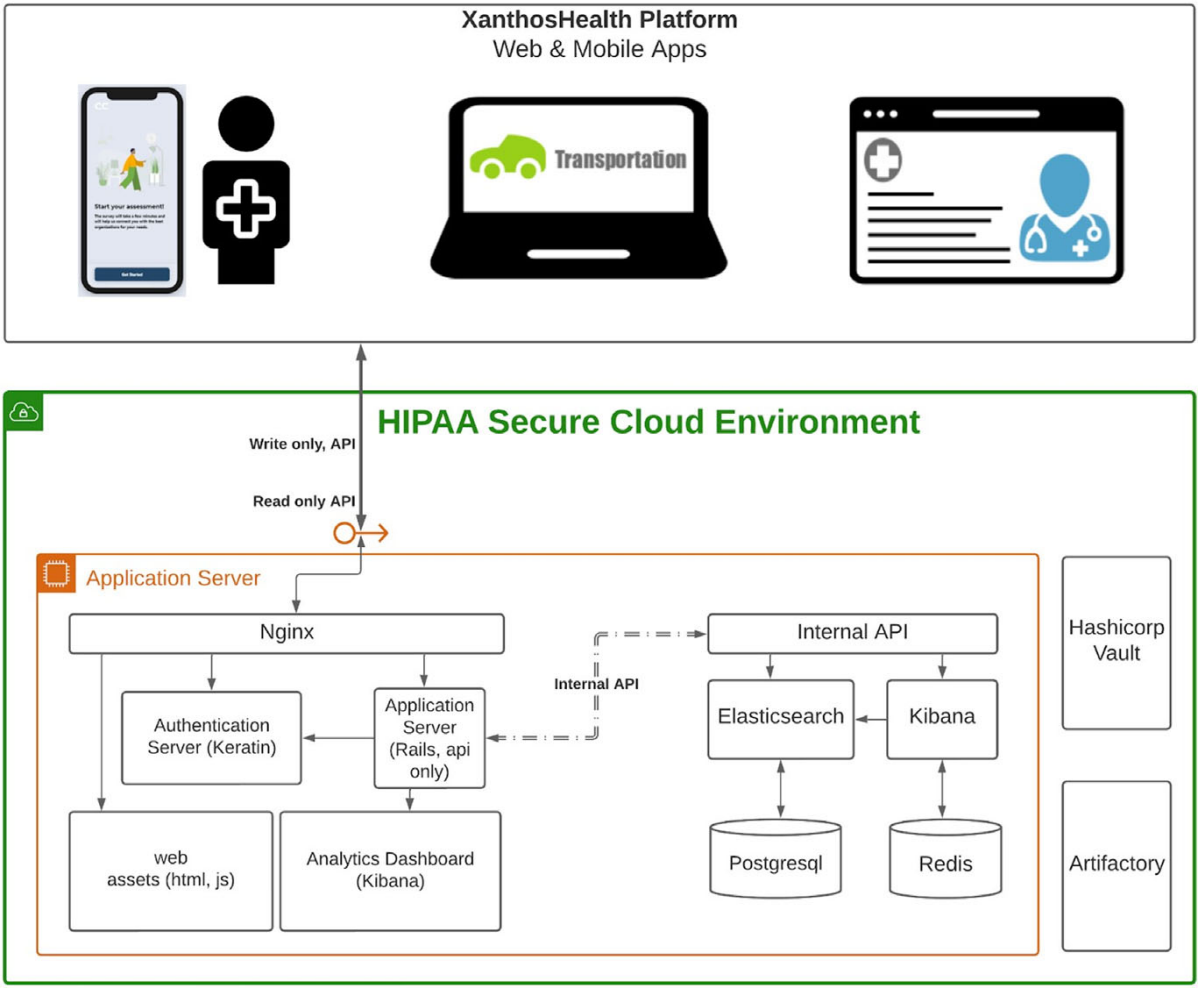
Overview of the ConnectedNest platform architecture. Legend: API: application programing interface; HIPAA = Health Insurance Portability and Accountability Act; HTML = HyperText Markup Language; JS: JavaScript.

ConnectedNest, through its Health Insurance Portability and Accountability Act (HIPAA) [31]-compliant data-sharing platform, connects individuals with social needs to CBOs through patient self-referral and/or oncology team referrals. The ConnectedNest architecture uses a modern web design approach for the multi-interface platform (Fig. 2). The user interfaces are built using React Native, which allows the patient interface to be developed and accessible on iOS and Android. The underlying architecture of the platform resides in a HIPAA secure environment that exposes two secure application program interface(s) used to communicate with public-facing interfaces. Ruby on Rails powers the platform and creates and maintains any changes within a PostgreSQL database. Elasticstack and its associated tools (i.e., Elasticsearch, Kibana, Beats, and Logstash) are used across systems for analytics and data retrieval. The prototype is EHR agnostic and has the capability to be used across multiple EHR platforms. The platform consists of three interfaces to support all three groups (patients, oncology care teams, and CBOs) to engage in the social-care referral process. ConnectedNest includes the following three components:

*1. EmpowerNest*

EmpowerNest is a patient interface that allows patients to self-screen for social needs and self-refer to organizations. ConnectedNest uses an algorithm to match patients to programs based on eligibility criteria. The patient-facing mobile app is designed to support self-screening for social needs and make referrals to CBOs. This self-screening, conducted on their own or at their clinic, can reduce stigma associated with direct questioning from clinic staff and increase access to resources. Assessments are written at a 4th-grade reading level. When individuals’ assessments indicate a need, they are immediately presented with organizations that provide services that can meet those needs for which they are eligible. Individuals can then choose which CBOs they would like to connect with. Patients can then indicate when a service was received.

*2. CommunityNest*

CommunityNest is the community organization web portal that allows community organization staff to create and maintain their community programs. The interface provides real-time descriptions of the status of referrals within the organization. CBO staff may also view patient needs and the status of existing referrals. The web portal allows CBOs to register their services. Once a CBO is registered, they are allowed to add and update their services and respond to clients who request services through the patient interface. It allows CBOs to specify and verify eligibility and assign eligible patients to team members so they can receive services as well as indicate when services were received. This portal also allows CBOs to refer patients to other CBOs within the platform.

*3. EngageNest*

EngageNest supports healthcare teams who are supporting their patients with an EHR interface. Healthcare teams can view patient needs and refer patients to CBO organizations that align with patient preferences. The EHR-enabled component of the platform allows oncology care teams (social workers, navigators, nurses, physicians, and others) to assist, advocate, and stay informed about the social-care referral. The “Substitutable Medical Applications and Reusable Technologies (SMART) on Fast Healthcare Interoperability Resources (FHIR)” [32] application allows the clinical care teams to be connected with their patients through the EHR. Clinical care teams can view the referral status and needs of their patients, receive status updates, and recommend organizations with whom their patients should connect.

**Figure 3.**
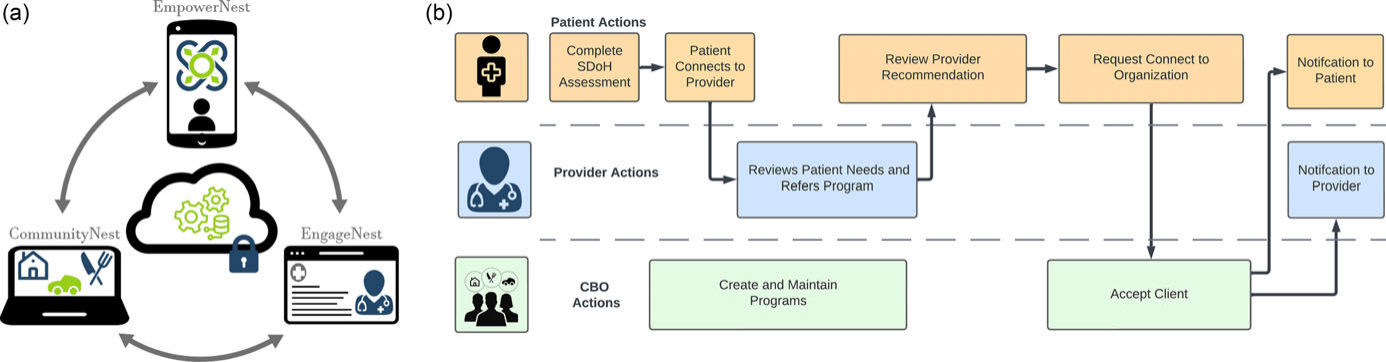
Overview of a) ConnectedNest components and b) an example healthcare system pathway for cancer patients, oncology care teams and community based organizations to address social needs after a cancer diagnosis. Fig. Legend: SDoH = social determinants of health; CBO = community based organization. SDoH = social determinants of health; CBO = community based organization.

ConnectedNest is designed to support multiple social need pathways, which may be initiated by each of our stakeholders (patient, oncology care team, and CBO). We describe a potential healthcare system pathway in Fig. 3. Figure 3 begins with a patient downloading the application on their own device during a clinical encounter. The patient completes the social needs screening assessment and then connects with their provider. This allows the provider to view patient’s needs and refer them to a CBO that matches their needs. Next, patients receive a notification on their device to review this provider-recommended CBO and the patient may choose to connect to the organization. CBO staff then receive a notification that someone has requested to use a particular program. CBO staff can review and accept that patient based on eligibility criteria. Accepted patients and providers are then notified when patients are accepted into a program.

### Prototype testing

#### Usability (UI/UX) testing

We conducted three rounds of task-based usability assessment with 16 individuals with cancer and their caregivers, 16 oncology care team members, and 16 CBO administrators throughout the design process. For each user interface (patient, provider, and CBO), we conducted three rounds of user testing. We followed the Lewis and James approach [33], which states that four to five user tests can detect 80% of a technology’s usability problems. Increasing the number of users has diminishing returns. For our technology, the first and second rounds had six users per interface, and four users per interface were used in the final round. Between each round of testing, problems identified by users were addressed and implemented in the prototype.

During patient interface testing (*EmpowerNest*), over 90% of users were able to complete basic tasks (i.e., registration, completion of a social needs assessment, and understanding their social needs). While patients reported interest in driving the social-care referral process, the interface initially lacked the appropriate information needed for patients to make decisions. In the final round of testing, 75% of users successfully connected to test organizations in the prototype.

Similar results were reported from the CBO testing interface (*CommunityNest*). Basic functions (i.e., organization creation, program creation, and chat) were accomplished by 90% of the users. However, the interface initially lacked the appropriate information for complex decision-making, such as evaluating if a client was eligible for a particular program and referring clients to other organizations. In our final round of usability testing, only one user was unable to complete both of these tasks.

The interface design for the clinical team (*EngageNest)* was similar to that of the CBO interface. Therefore, much of the design learnings of the CBO interface could be applied to the EHR interface. Unlike the CBO staff, 80% of clinical team users were successful in referring patients to CBOs. Clinical workflow and the visual design of the interface initially made it difficult for clinical team members to work with an external system. For example, initially, only 60% were able to “add a patient” to their clinical team and understood that context. Based on this feedback, additional modifications were made and, in our final round of testing, only one user was still unclear about the process for adding patients.

## Discussion

Given the dramatic growth in the financial burden of cancer over the past decades, cancer patients are at particular risk for social needs and experiencing an adverse impact of these needs on care management and health outcomes. Using input from a diverse set of focus groups and KI participants, user experience/user interface testing, and a multidisciplinary community advisory board, we developed a new technology solution, ConnectedNest, which connects individuals in need to CBOs through direct and/or oncology team referrals, with interfaces to support all three groups (patients, CBOs, and oncology care teams). After prototype development, we conducted usability testing, with broad success (>80%) for participants to complete key tasks within the prototype. Throughout the development process, participants noted the importance of technology like ConnectedNest to fill current gaps in screening and connection of individuals with cancer with needed social and community services using a patient-empowered approach with the support of their healthcare team and community organizations.

The importance of addressing social needs among individuals with cancer will only grow with the rising costs of cancer care and shift toward incorporating social needs metrics into models of value-based care [5,8]. Using multi-stakeholder input, ConnectedNest provides an opportunity to address these needs by providing a cohesive ecosystem that allows all stakeholders to engage. We prioritized ConnectedNest to be centered on patient empowerment, putting access to social services at the fingertips of users by giving them an active role in their health. In addition, we wanted to create a system in which CBOs could easily engage by listing and updating their services, including real-time updates such as pop-up services or traveling kitchens. Flexible solutions like ConnectedNest have the potential to be adapted and incorporated into a diverse set of health systems and community organizations. The innovative EHR-enabled health information technology platform allows for screening, assessing social needs, and connecting individuals with cancer to CBOs that can address their needs with the overall goal of improving the health and psychosocial outcomes of these individuals.

Our multi-stakeholder engagement with patients, oncology care teams, and CBOs revealed a need for flexible, capacity-enhancing solutions for a system that is resource-constrained. Oncology care teams use a variety of social needs screening tools to evaluate social needs but have limited staffing and time available [7]. They also utilize a variety of workflows in order to screen and connect individuals to needed services [7]. CBOs have evolving programing to address the needs of their clients’ and patients’ needs that will necessarily change throughout the course of their diagnosis, treatment, and survivorship. Recognizing the need for a flexible technology, we directly addressed these hurdles by building an adaptable technology that can fit the needs of a diverse set of organizations, allowing for: 1) use of a variety of social needs screening tools, 2) EHR-enabled technology with the ability to work across a diverse set of oncology care workflows, 3) providing direct access to CBOs to create up-to-date programing, and 4) real-time evaluation of current needs for patients throughout the trajectory of their diagnosis.

Overall, this work has highlighted the challenges with the current system for screening and referral for social and community services. Prior work, which is supported by our focus groups and KI interviews, highlights that traditionally, connecting individuals with resources to address social needs has fallen to patient navigators and social workers within the oncology care teams [34]. However, like many multidisciplinary oncology care teams in the United States, the resources needed to connect individuals with needed social and community services exceed the available staffing [35]. There is a critical need to identify solutions to build capacity for more effectively connecting oncology patients with needed social and community resources to alleviate distress. Cancer is a unique disease that is highly time and resource-intensive, requires ongoing collaboration with multidisciplinary care teams throughout treatment, and, given treatment advances, has expanded the scope and trajectory of survivorship. This unique combination calls for a unique approach to connection with social and community services that is patient-empowered and goes beyond existing technologies.

With the development of the ConnectedNest prototype, future research will examine the use and integration of this technology in health systems and use among other stakeholders (e.g., payers). Specifically, in September 2022, XanthosHealth participated in the Sync for Social Needs Initiative [36], announced by the White House, uniting leading health technology companies and health systems to standardize the sharing of patient data on social needs, including food insecurity. Currently, XanthosHealth and University of Minnesota researchers are conducting a real-world pilot in the Twin Cities, Minnesota with the Minnesota Cancer Alliance (MCA) [37], a convener of over 100 cancer-specific organizations. This work builds on the engagement with CBOs during the prototype development phase through Community Advisory Board meetings, KI meetings, and usability testing supported through a National Cancer Institute-funded Phase I Small Business Innovation Research (SBIR) project award. In the current pilot, the team is engaging with CBOs that are part of the MCA, and conducting KI interviews to understand how to best meet the social needs of individuals with cancer through ConnectedNest. The study is also recruiting patients directly from CBOs that are part of the MCA network to pilot test ConnectedNest, as well as working directly with a select group of CBOs for a deeper understanding of the challenges and necessary software and workflow enhancements to impact patient-centered outcomes in this population. Future phases of ConnectedNest development will include integration of the technology into the electronic medical record, which is supported by the current SMART on FHIR design. As part of this development, we plan to include discussions on how and where data are stored as well as patient notification processes for oncology care team members to access this information. We are additionally examining how best to engage patients and CBOs to ensure that included services are up-to-date and effective. This work can be used to inform initiatives such as payer-driven value-based reimbursement initiatives and US Centers for Medicare & Medicaid Services demonstration projects [38]. As part of these discussions, we must ensure stakeholders are engaged to provide feedback on the incorporation of tools such as ConnectedNest to ensure sustainability of these technologies coupled with development and expansion of resources to meet the social needs of the communities. At the same time, we must ensure that as efforts to screen for social needs become increasingly integrated into the clinical setting [39], new tools support alignment of reporting on these needs in order to reduce duplicate information and burden on the care team. ConnectedNest works toward this alignment by placing resources and services in a single tool, with one set of registration information accessible in the same format to participating community organizations.

We recognize certain limitations with the current work. First, our focus group and KI interviews were focused on English-speaking cancer patients, providers, and CBOs based in a single, large metropolitan area. While we focused on recruiting a diverse population of stakeholders with varied backgrounds and experience, and whose experience aligned with barriers noted in previous literature, our study population was not as socio-economically diverse as the US population in terms of racial/ethnic composition, gender, and income. Future studies can examine additional opportunities for addressing social needs with technology solutions among broader cancer populations with additional focus on socio-demographically underrepresented populations. Additionally, we conducted our work when many health systems were operating under constrained resources and modified protocols during the COVID-19 pandemic. While operations and staffing may eventually return to pre-COVID levels, the insights provided by all stakeholders in the development of the prototype allow us insight into how technology like ConnectedNest might provide benefits at the height of health system and financial constraints. Importantly, we must ensure that programs and funding developed and implemented to address social needs during the height of the pandemic remain sustainable, as the effectiveness of tools like ConnectedNest can only be maximized if CBOs have adequate resources and support for their programing. Finally, while our prototype was developed to promote usability and accessibility across a broad range of patient populations, including use of screening tools written at the 4th-grade reading level, future expansions of this work should ensure that tools like ConnectedNest are accessible for individuals with limited health literacy, those with limited English proficiency, and those with limited access to digital technologies.

Going forward, future work will examine the integration and implementation of ConnectedNest for oncology patients, oncology care teams, and cancer-focused CBOs to build capacity for effectively addressing distress in this population. This solution has the potential to provide an innovative, cost-effective, sustainable, and scalable way to enhance capacity to connect individuals to social and community services across other cancer centers and practice settings in the US.

## Supporting information

Parsons et al. supplementary material 1 Parsons et al. supplementary material

Parsons et al. supplementary material 2 Parsons et al. supplementary material

Parsons et al. supplementary material 3 Parsons et al. supplementary material

## Supplementary material

The supplementary material for this article can be found at https://doi.org/10.1017/cts.2024.570.
