# Supplementary material for: Addressing social needs in oncology practices: A case study of a patient-centered approach using health information technology
Source: J Clin Transl Sci. 2024 Sep 30;8(1):e139. doi: 10.1017/cts.2024.570 (PMC11523020; doi:10.1017/cts.2024.570)
Supplement: Parsons et al. supplementary material 1 — Parsons et al. supplementary material [file S2059866124005703sup001.docx]

**Supplemental Table 1. Focus Group Discussion Guide**

| 1. | Let’s start by going around and introducing ourselves:   - What do you want to be called today? *[an alias or initials rather than your name if you would like].* - Say one thing you’d like us to know about you. - Share what you use most on your phone (like your favorite app); or what technology you use most. |
| --- | --- |
| 2. | Thinking about your experience receiving or supporting someone who was receiving care for cancer, what types of non-medical services or supports would have been useful for you to know about? Which of these services are or would have been most important to you?  Prompt: Can you tell me more about that? Can you tell me what you mean by that? |
| 3. | Thinking about the non-medical services and supports that may be important to you or other cancer survivors, what would be the ideal way for you to identify an interest in receiving these services? Would you be willing to complete a short survey to indicate your interest in these services? When and/or how often?  Prompt: I notice you haven’t said anything about:   - Preferences for paper/online? - Preferences for length? - Data security concerns? |
| 4. | Do you see any challenges to accessing services you may need or be interested in? Are there ways that might make it easier for you to access these services?  Prompt: Can you tell me more about that? Can you tell me what you mean when you say you [that]? Why do you think it’s a challenge? |
| 5. | How would you like to be connected to needed services or supports? If you could change the way that you and others like yourselves are connected to services they need in a way that is the most helpful as possible for you, what would that look like? |
| 6. | If you could change the way that you and others like yourselves are connected to services they need in a way that is as helpful as possible for you, what would that look like?  Prompt: I notice you haven’t said anything about:   - Who will be involved (oncology care team)? - Use of technology? - Data security? |
| 7. | Is there more you would like to add? Anything you feel was missed in this discussion? |
