# Supplementary material for: Addressing social needs in oncology practices: A case study of a patient-centered approach using health information technology
Source: J Clin Transl Sci. 2024 Sep 30;8(1):e139. doi: 10.1017/cts.2024.570 (PMC11523020; doi:10.1017/cts.2024.570)
Supplement: Parsons et al. supplementary material 2 — Parsons et al. supplementary material [file S2059866124005703sup002.docx]

**Supplemental Table 2. Key Informant Interview Guide**

| 1. | First, I want to start by asking about your role in your organization, the title of your position.  What is your training/background? E.g., physician, nurse, social worker, administrator, etc? Does your position and/or responsibilities involve either connecting individuals with cancer with needed social and community services or providing those services? If so, how? |
| --- | --- |
| 2. | In your system or organization, how do you identify individuals who may need social or community services? How is this information stored and/or shared both within/outside your organization?  Prompt: I notice you haven’t mentioned anything about   - Collection tools (e.g., EPIC, paper) - Data security/sharing |
| 3. | A number of validated screening tools exist to screen individuals for needed social and community supports, some cancer-specific and some for the general population. Examples include the Distress Thermometer, the EveryONE Project and PRAPARE tool (provide a copy of example screening tools for reference). Are there specific screening tools or instruments you use or think would be most useful to identify these individuals? What needs should they screen for and/or prioritized? Are there specific thresholds or indicators you use to identify someone as ‘needing’ a specific social and community services? |
| 4. | Can you describe the general process for how individuals are connected to needed social and community services once their need is identified? What role do you and/or your organization play in this process? Who is involved?  Prompt: I notice you haven’t mentioned anything about:   - Staff/personnel involved - Paid vs. volunteer and level of effort |
| 5. | What works well under this current system? What are challenges you see with the current system for screening individuals for needed services and connecting them to those services? |
| 6. | If you could design an ideal tool or process to ensure patients had access to needed community and social services, what would it look like?  Prompt: I notice you haven’t mentioned anything about: Data collected/reported, etc. |
| 7. | What role could a mobile application ideally play as part of this ideal process? What aspects of the screening, identification and connection should be included in the mobile application? What information would you want this application to have to be of most use to you in your work with oncology patients? |
| 8. | Is there anything else you would like to add? Anything you feel was missed in this discussion? |
