# Supplementary material for: Addressing social needs in oncology practices: A case study of a patient-centered approach using health information technology
Source: J Clin Transl Sci. 2024 Sep 30;8(1):e139. doi: 10.1017/cts.2024.570 (PMC11523020; doi:10.1017/cts.2024.570)
Supplement: Parsons et al. supplementary material 3 — Parsons et al. supplementary material [file S2059866124005703sup003.docx]

**Supplemental Table 3: Community Advisory Board Members**

| Organization | Description |
| --- | --- |
| Angel Foundation  [https://mnangel.org](about:blank) | Twin cities based, offers emergency financial assistance for non-medical expenses (e.g., rent, cell phone, gas/electric bills, food) for adults undergoing active cancer treatment. |
| Breast Cancer Education Association (BCEA)  [https://breastcancereducation.org](about:blank) | Mission is to educate the community about breast cancer while also providing support |
| American Cancer Society  [https://www.cancer.org](about:blank) | Provides detailed information on cancer as well as how to maintain healthy lifestyle. In addition, their programs such as the “Road to Recovery” program provides rides to and from cancer-related medical appointments for patients who otherwise might not be able to get there. |
| Sage Screening Program  [https://www.health.state.mn.us/diseases/cancer](about:blank)  [/sage/services/index.html](about:blank) | Provides screening for underinsured/uninsured Minnesota women |
| Firefly Sisterhood  [www.fireflysisterhood.org](about:blank) | Nonprofit program that matches women experiencing breast cancer with breast cancer survivors |
| Gilda’s Club  [https://www.gildasclubtwincities.org/who-we-are/](about:blank) | Offers free, social, psychological and emotional support for anyone impacted by cancer (virtual options) |
| Cancer Legal Care  [https://www.cancerlegalcare.org/](about:blank) | A Minnesota non-profit of staff and volunteer attorneys who provide free direct legal care services to address the employment, disability, estate planning, financial, and housing issues to cancer patients |
| Cancer Health Equity Network  [https://mncanceralliance.org/chen/](about:blank) | A collaborative organization of community health workers, community-based organizations, and cancer professionals, who work to inform the community of cancer concerns. |
| Second Harvest Heartland  [https://www.2harvest.org/](about:blank) | Addresses food insecurity through a network of 816 food shelves, meal programs, food distributions, discount grocery stores in all 87 Minnesota counties. |
| American Indian Cancer Foundation  [https://www.americanindiancancer.org/](about:blank) | Addresses cancer inequities faced by Native communities through improved access to prevention, early detection, treatment, and survivor support. |
| A Breath of Hope Lung Foundation  [https://abreathofhope.org/](about:blank) | Provides support services for those living with lung cancer to lighten their burden and fill in the gaps where additional support is needed. |
| Be the Match  [https://bethematch.org/](about:blank) | Provide one-on-one support to patients and their families every step of the way along the transplant journey. |
